# Supplementary figures and images for: Adverse health correlates of intimate partner violence against older women: Mining electronic health records
Source: PLoS One. 2023 Mar 8;18(3):e0281863. doi: 10.1371/journal.pone.0281863 (PMC9994723; doi:10.1371/journal.pone.0281863)

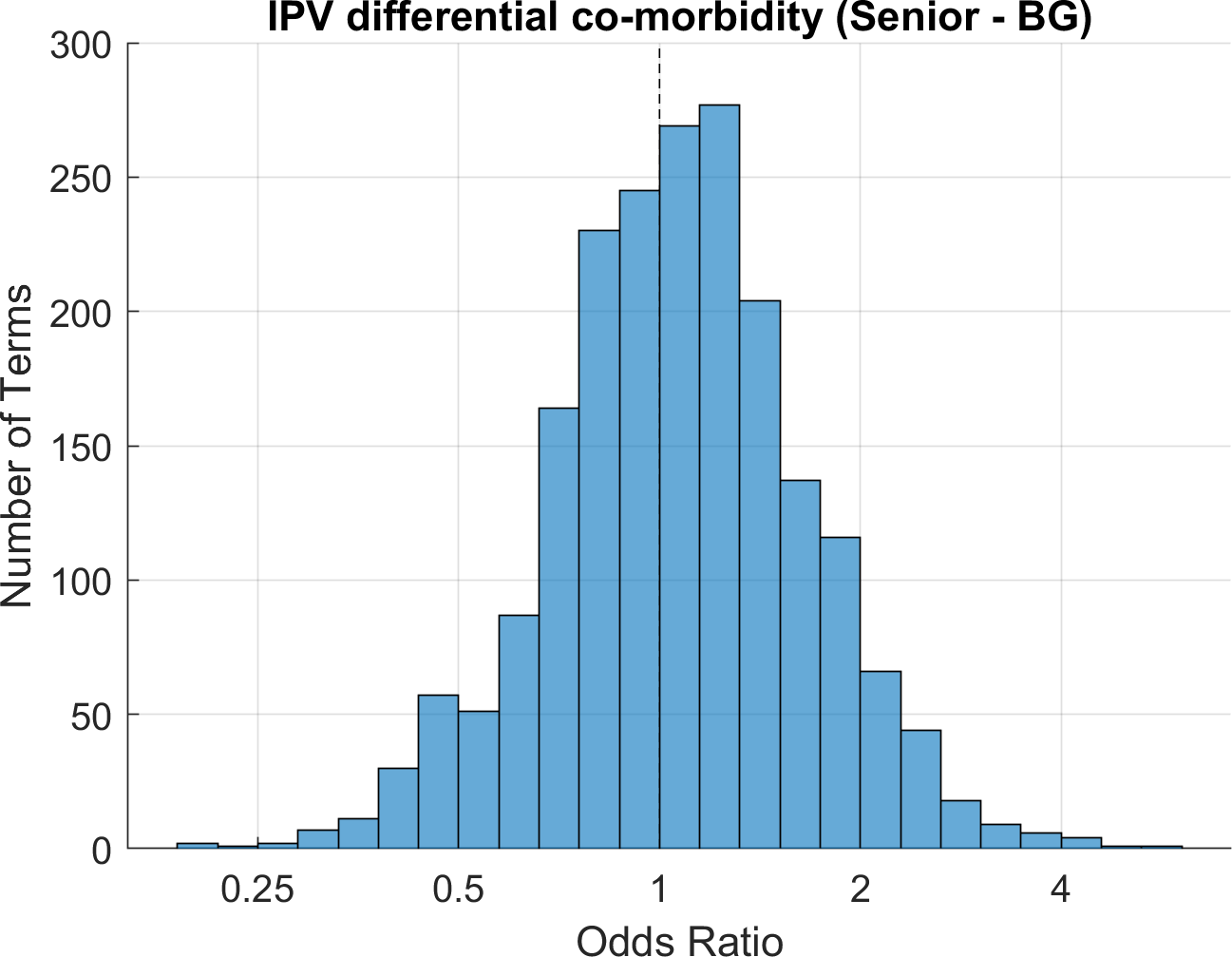

Supplement: S1 File — (ZIP) [file pone.0281863.s004.zip › IPV_Older_Women/out/senior/Ever/Figures/histogram_differential_comorbidity_marked.png]

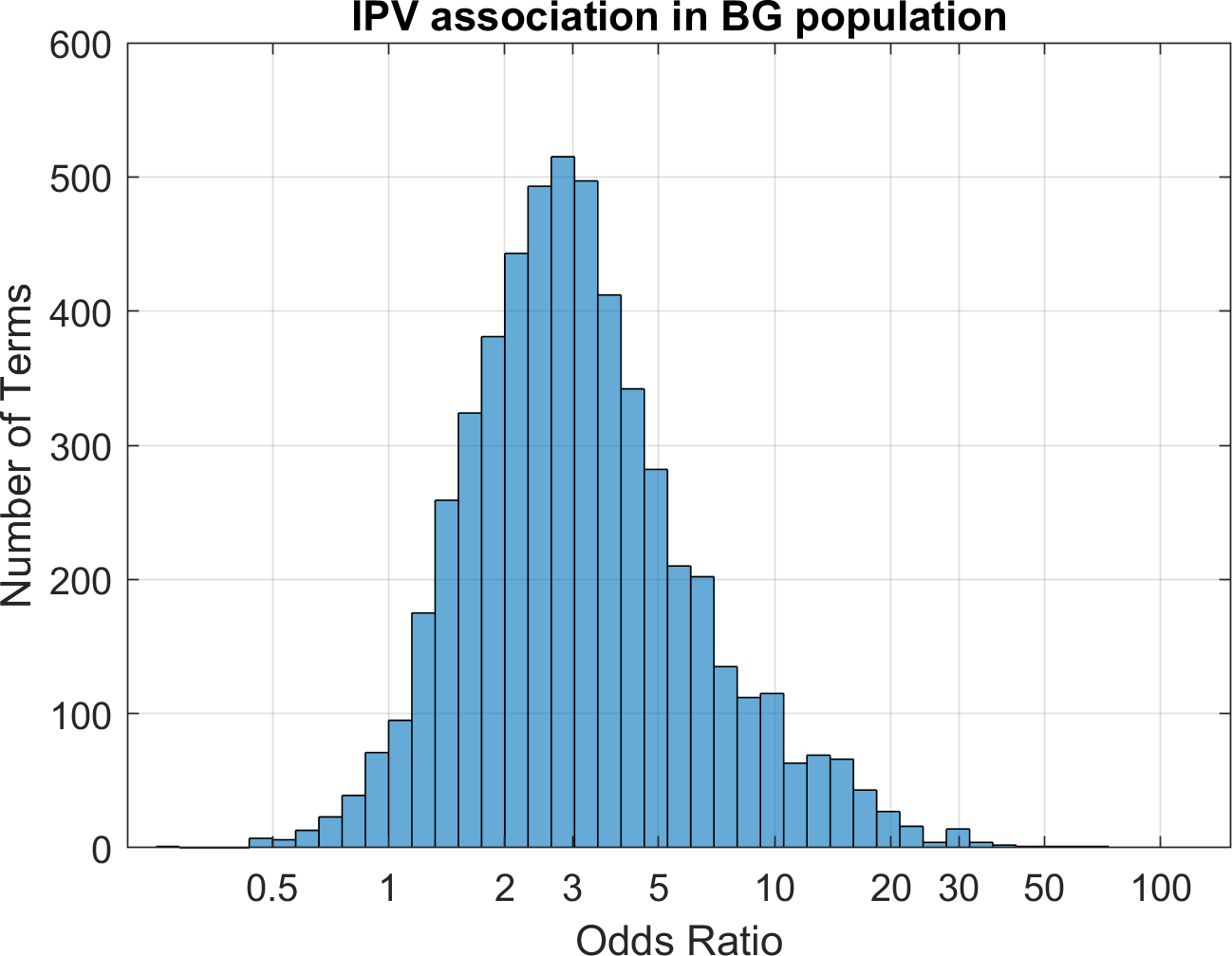

Supplement: S1 File — (ZIP) [file pone.0281863.s004.zip › IPV_Older_Women/out/senior/Ever/Figures/histogram_ipv_bg.png]

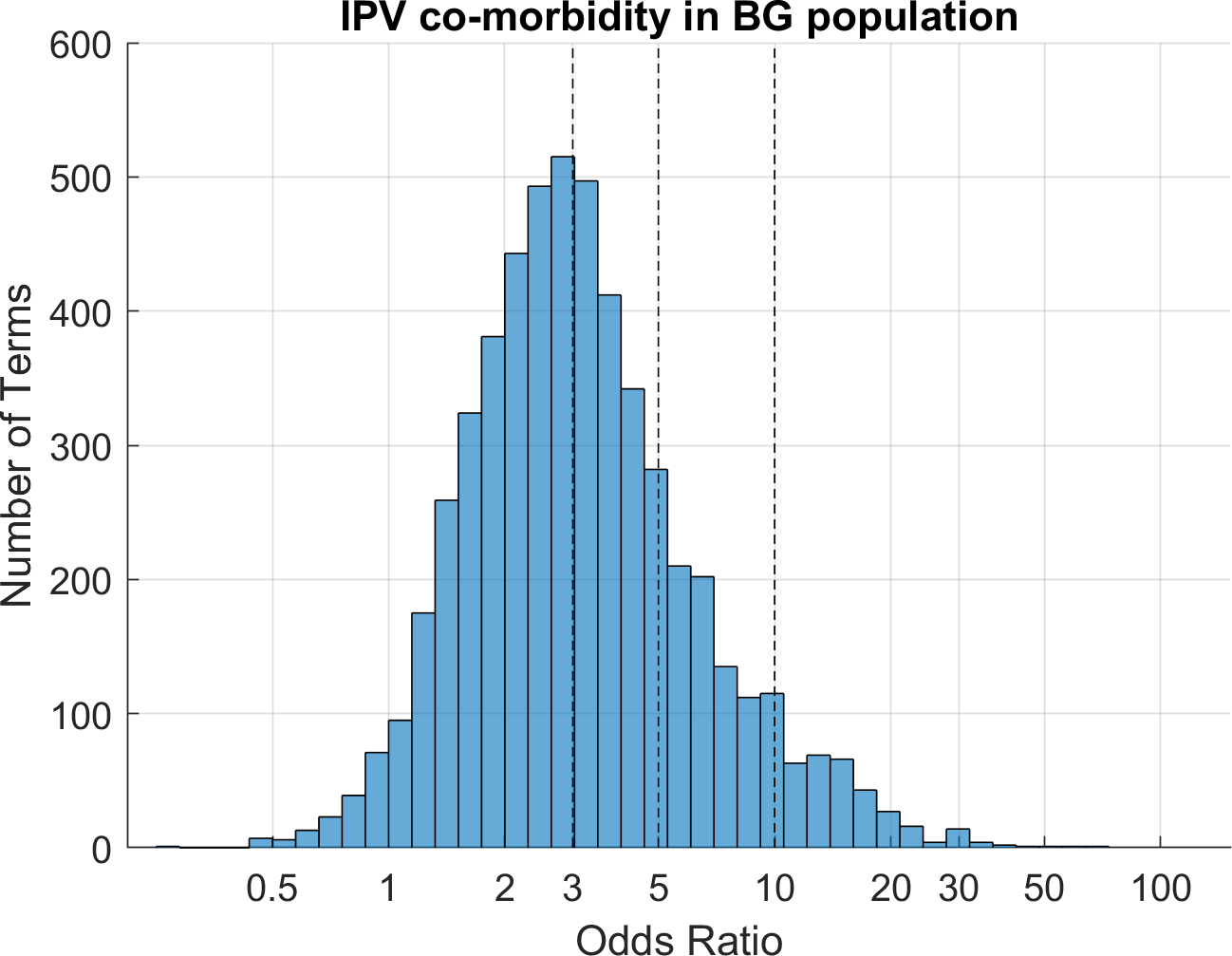

Supplement: S1 File — (ZIP) [file pone.0281863.s004.zip › IPV_Older_Women/out/senior/Ever/Figures/histogram_ipv_bg_marked.png]

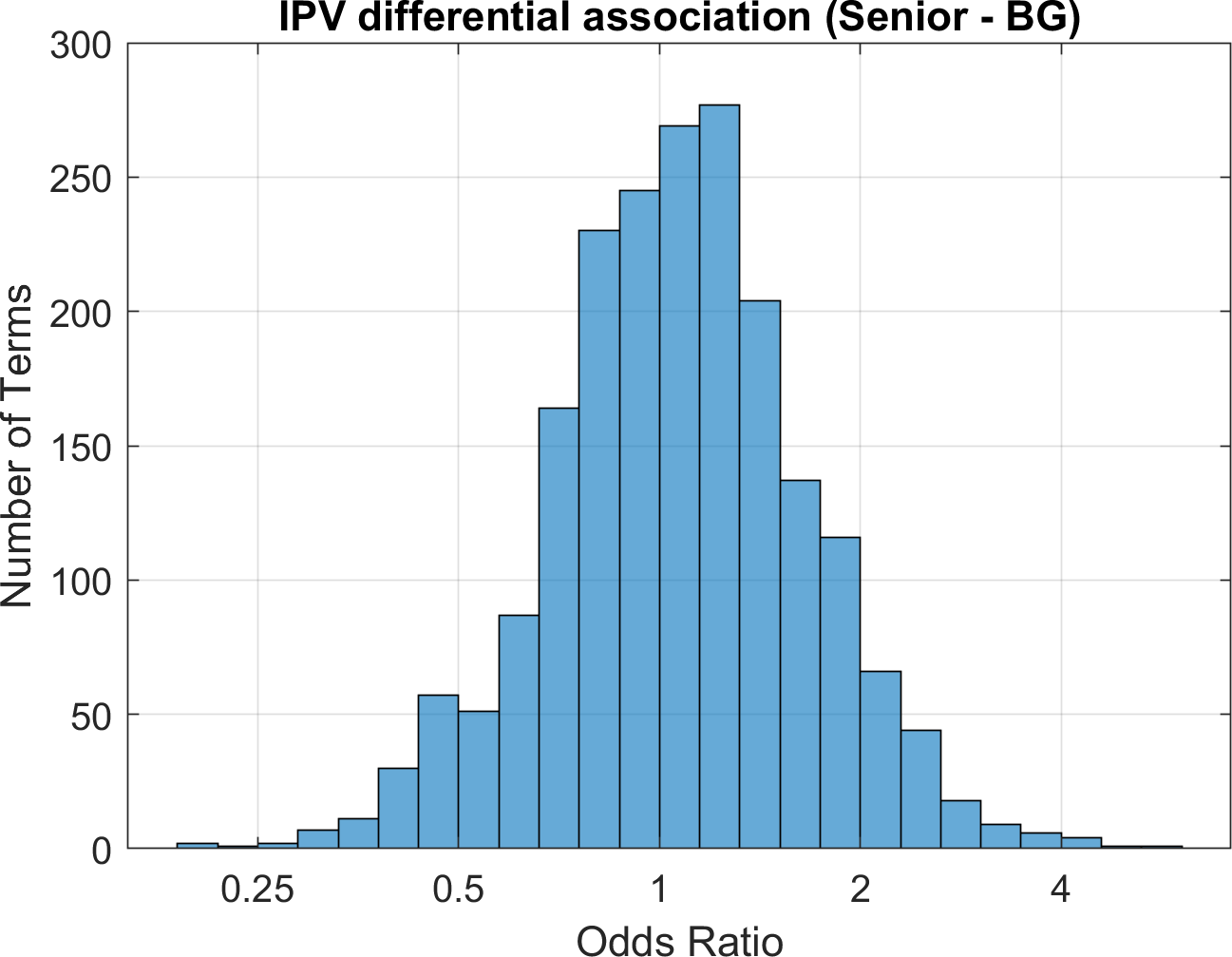

Supplement: S1 File — (ZIP) [file pone.0281863.s004.zip › IPV_Older_Women/out/senior/Ever/Figures/histogram_ipv_differential_senior_bg.png]

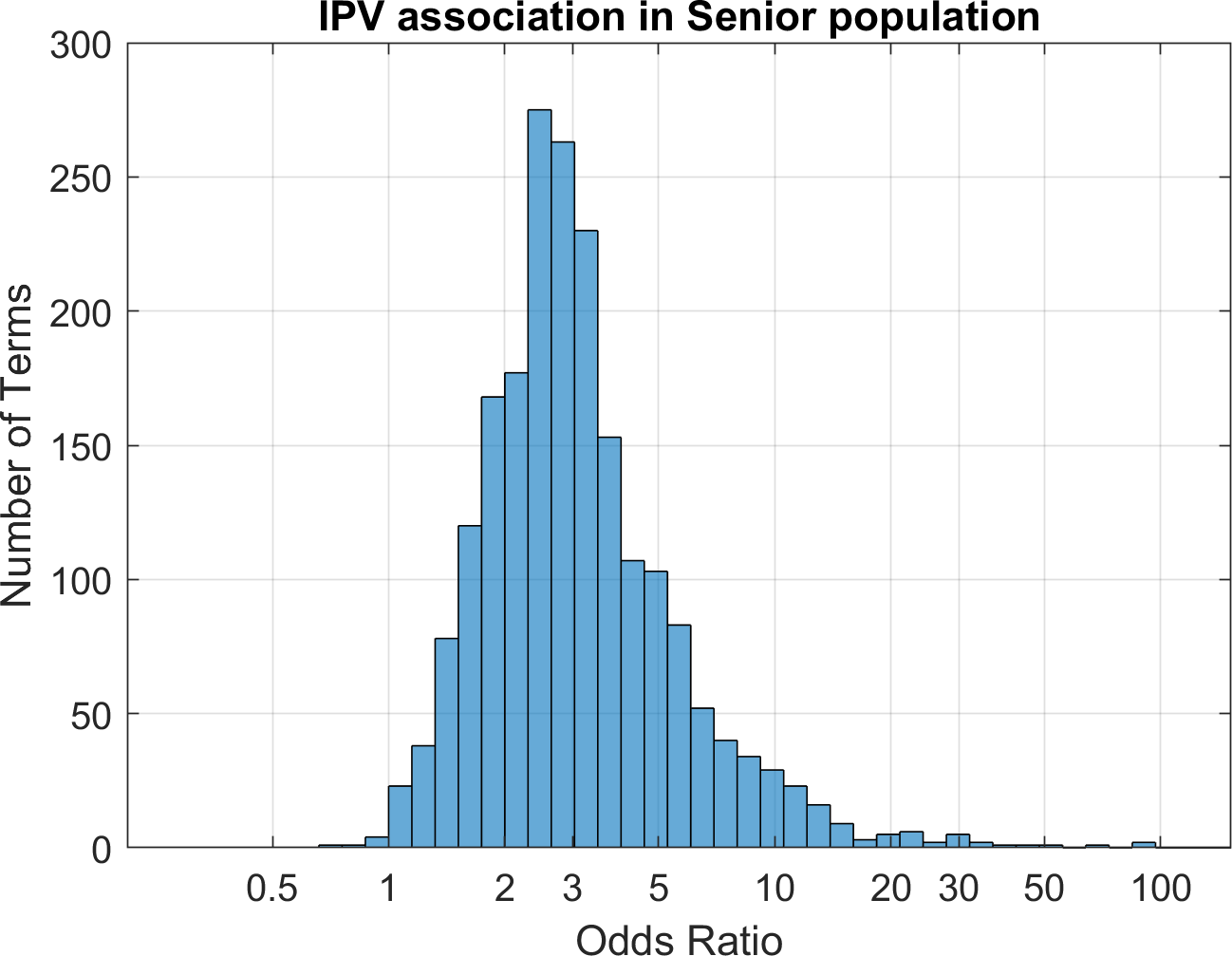

Supplement: S1 File — (ZIP) [file pone.0281863.s004.zip › IPV_Older_Women/out/senior/Ever/Figures/histogram_ipv_senior.png]

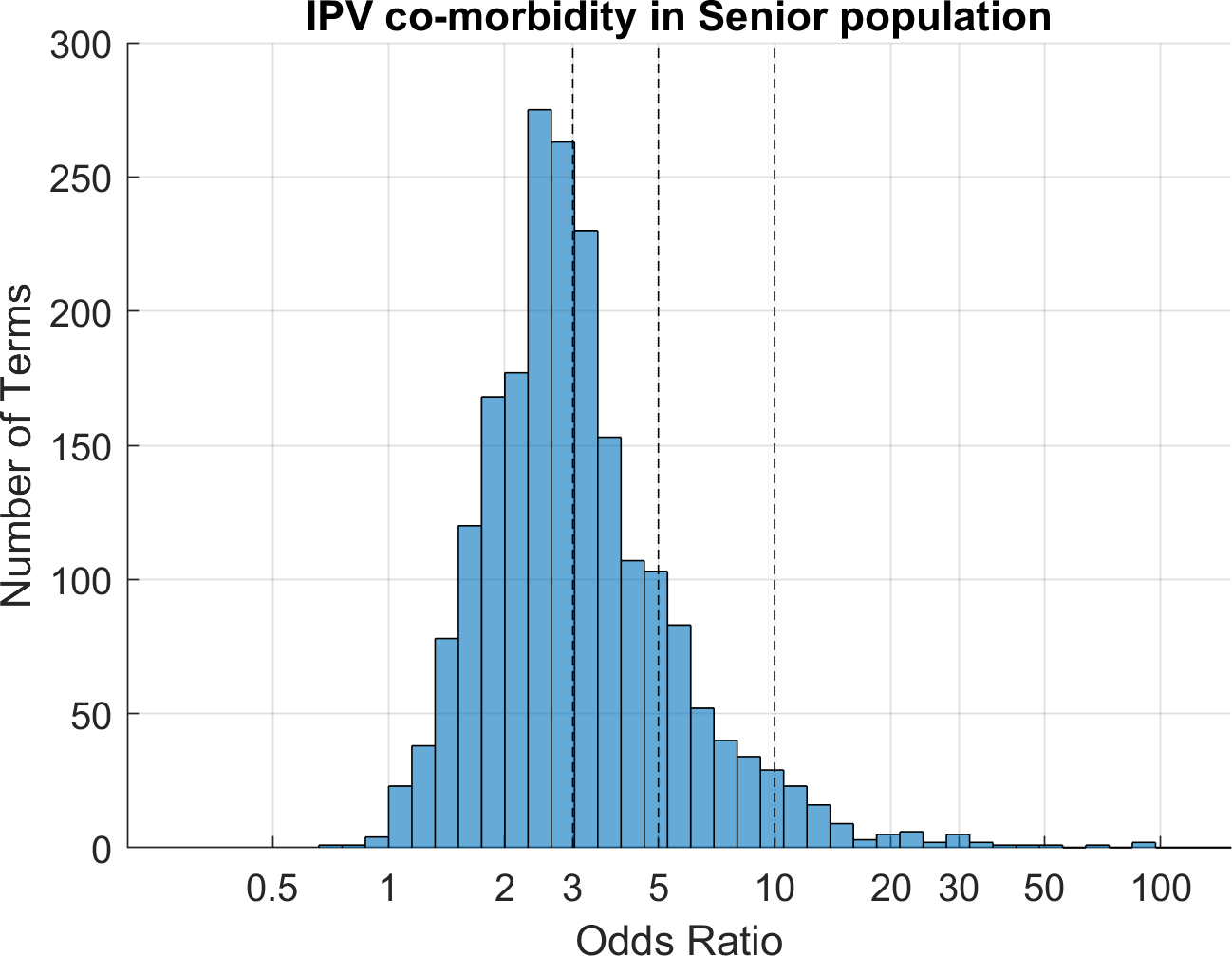

Supplement: S1 File — (ZIP) [file pone.0281863.s004.zip › IPV_Older_Women/out/senior/Ever/Figures/histogram_ipv_senior_marked.png]

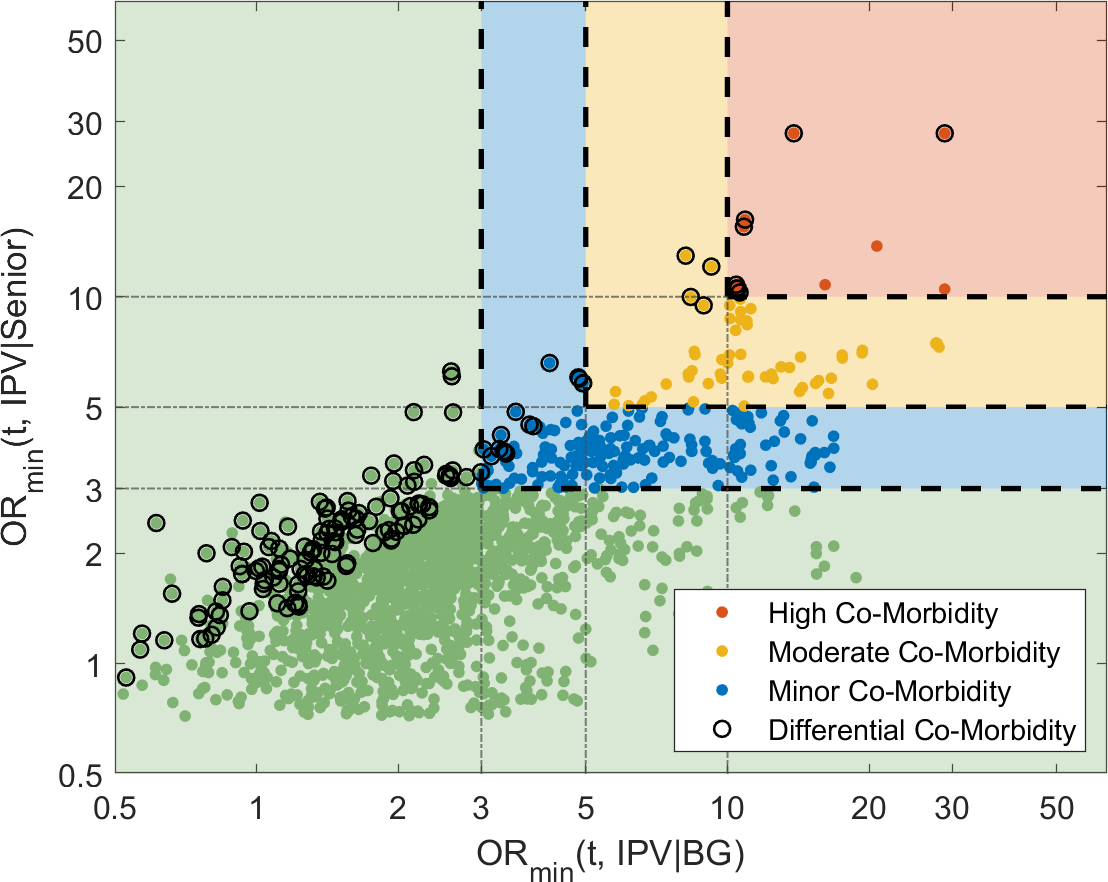

Supplement: S1 File — (ZIP) [file pone.0281863.s004.zip › IPV_Older_Women/out/senior/Ever/Figures/ipv_senior_regionplot.png]

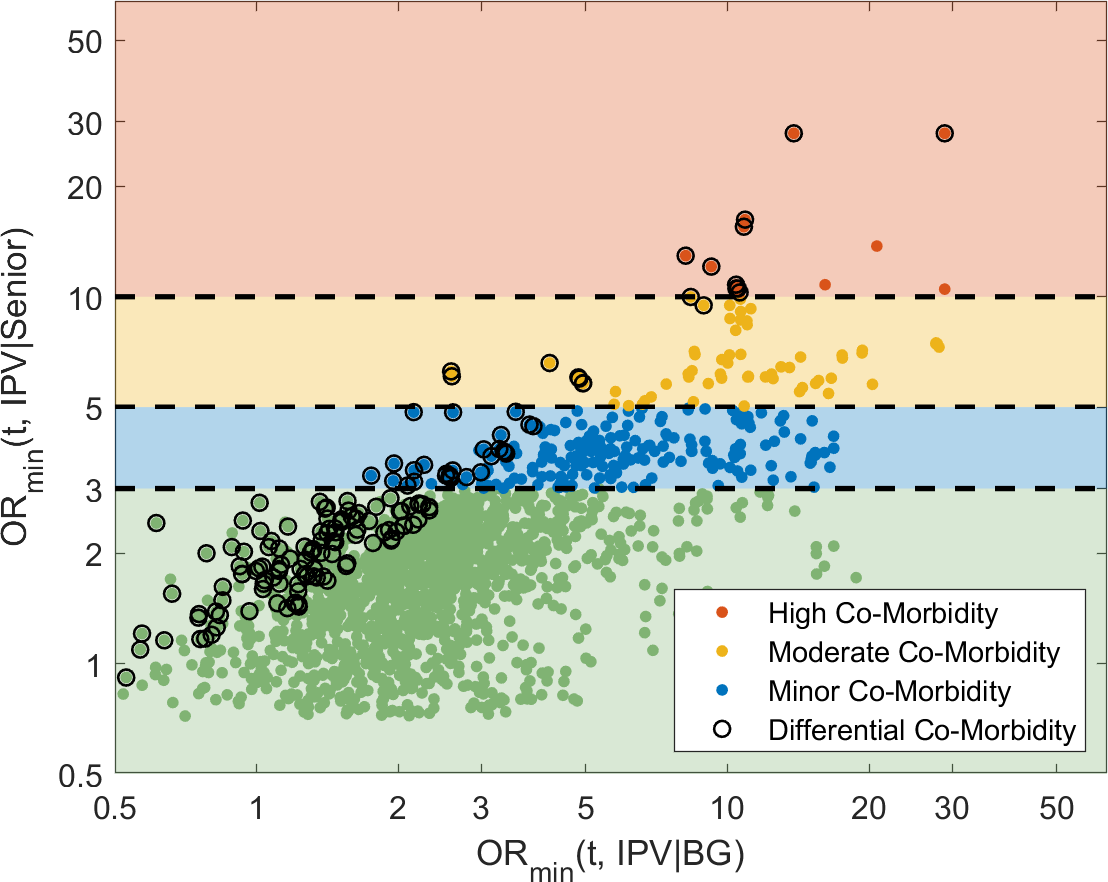

Supplement: S1 File — (ZIP) [file pone.0281863.s004.zip › IPV_Older_Women/out/senior/Ever/Figures/ipv_senior_regionplot_alt.png]

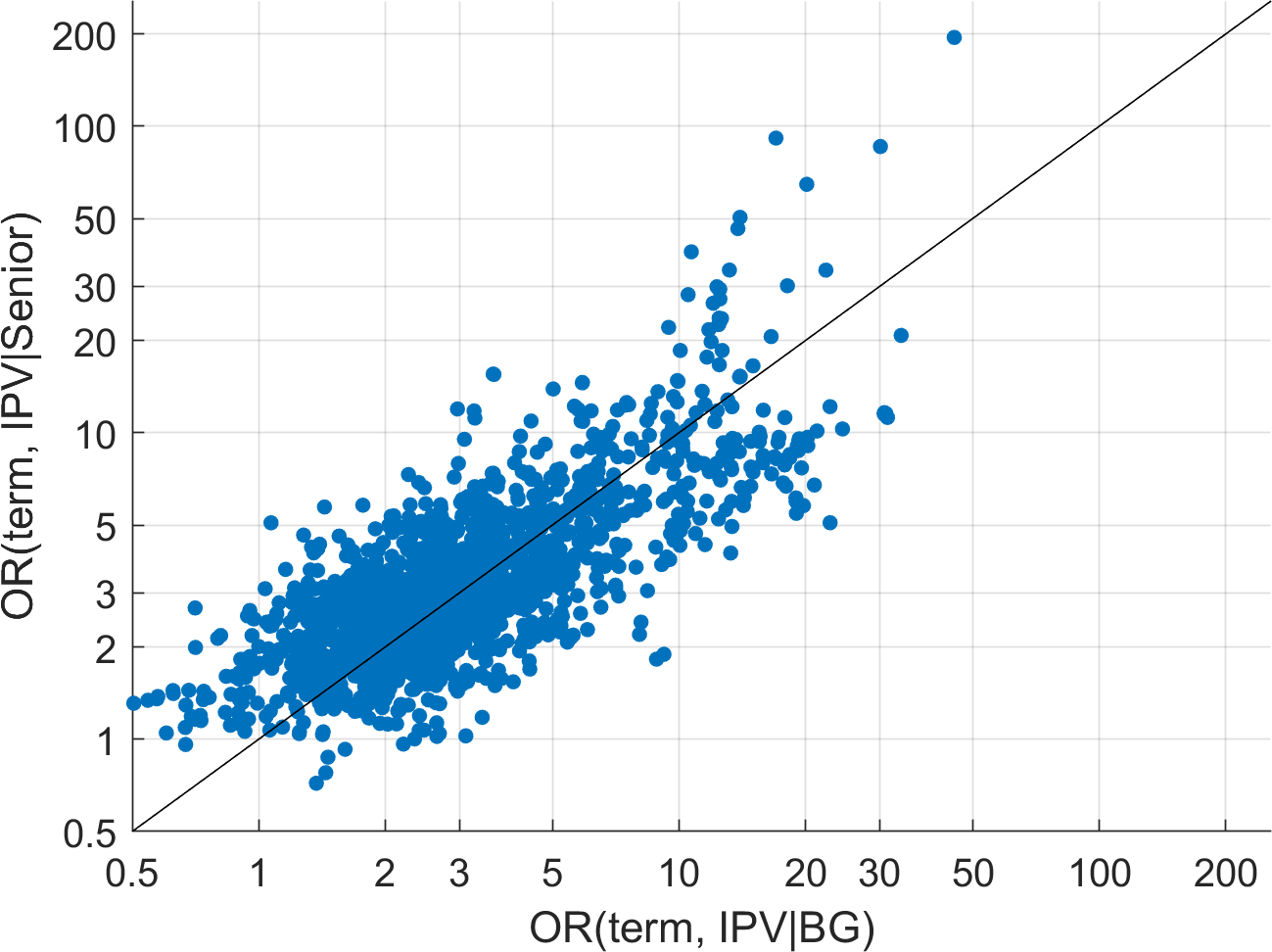

Supplement: S1 File — (ZIP) [file pone.0281863.s004.zip › IPV_Older_Women/out/senior/Ever/Figures/scatterplot_senior_vs_bg.png]
