# Supplementary material for: Adverse health correlates of intimate partner violence against older women: Mining electronic health records
Source: PLoS One. 2023 Mar 8;18(3):e0281863. doi: 10.1371/journal.pone.0281863 (PMC9994723; doi:10.1371/journal.pone.0281863)
Supplement: Attachment — Submitted filename: ipv_older_women_response_letter_round_2.pdf [file pone.0281863.s007.pdf]

Adverse Health Correlates of Intimate Partner Violence against Older Women: Mining Electronic Health Records

Reviewers' comments:

1. First, the study results are interesting. However, there is significant room for improvement regarding the appropriateness of the scientific method in the publication criteria of PLOS ONE.

   We would like to thank the reviewers for their feedback to improve the manuscript and the scientific methods.

2. I agree with comment 1 of the previous Rev #1. In fact, many formulas can be omitted. For example, the formulas for calculating crude odds ratios and 95% confidence intervals from a 2 x 2 contingency table can be found in regular textbooks (e.g., equations 1, 2, 3, etc.), so there would be no need to include them in the text. Since PLOS One is a journal with readers from various educational backgrounds, please explain in writing what you have done unless it is absolutely necessary to use mathematical formulas. Since this paper is not a paper on statistical methods, writing formulas would rather spoil the merit of this paper. This applies to other parts of the method as well. If the authors want to write the formulas described in Revision 1, upload them as Supplemental Material.

   We would like to thank the reviewer for bringing this issue back to our attention. Although it seems like the formulas are unnecessary to add to the paper, indeed there are some nuances and revisions and it is critical that we add those in there, so that the unique approach that helps us to answer questions related to EHR databases can be utilized by other researchers. EHR databases have great potential to discover patterns of various health concerns. However, there are not many papers in the literature detailing the specifics of how this kind of analysis can be carried out. We have put a lot of thought and given great attention to details to uncover these patterns. We carried some of the formulas to the supplementary document while keeping the others that we think are necessary to keep in the manuscript for the completeness of the framework.

3. As mentioned in response to Rev #1, there are differences in the results from different statistical software, or discrepancies in the calculation results due to different versions of the software, even within the same software. Therefore, the code or program used by the authors (in C or any other language, e.g. R code) itself should be uploaded as Supplementary Material.

   Thank you so much for this comment. Exactly for this reason, we would like to add the formulas, and our conceptual framework into the paper. As PlosOne has readers from various educational backgrounds, we believe that methodological contributions that we make here, will be well received among the clinical informatics community. We uploaded the source code of our analysis in Matlab as Supplementary Material.

4. The censoring that the authors refer to would be better described as rounding or rounding off. Censoring, as usually used in cohort studies, usually refers to a participant dropout or exceeding the upper measurement limit for counts or measurements. Please reconsider your use of the term. Also, please remind us in the methods section that rounding occurs in the dataset.

   Thank you for helping us to make this clarification. To address this issue, in the revised manuscript, we now use the term rounding instead of censoring to refer to the rounding of the counts.

5. There needs to be a more detailed written explanation of how the Monte Carlo simulations were performed. Also, to what extent would incorporating this procedure change the results in the first place? Sometimes it is more accurate to dare to do nothing. At a minimum, the authors need to cite papers that use similar methods and claim their effectiveness. They should also show the results of bypassing this procedure as a sensitivity analysis to demonstrate the effectiveness of this procedure. If there is no significant change (i.e., the number of words extracted is zero or doubled), then it would be simpler and better to omit this process. For example, suppose the height is 171.8 cm. Here, the true value would be between 171.75 and 171.85 cm, but one would not normally use the Monte Carlo method in analyzing such data; as the number n increases, one would expect the overall rounding error to average out (law of large numbers).

   This is an excellent comment. We thank the reviewer for expressing this concern. The reviewer is correct in that the rounding does not have a major effect for large numbers. However, since the frequencies of terms are quite variable in our analyses, we thought this effect could be important for terms that are less frequent. As the reviewer predicts, the effect of this procedure on the top findings are minimal (in the sense that the top 20 terms identified as significant in Table 1 and 2 are also significant if this procedure to account for the rounding is not performed). However, this procedure has a considerable effect on the following:

   - (1) It suppresses possible false positive errors that are due to the infrequent terms having low number of records overlapping with IPV (e.g., terms exhibiting only 10 records having IPV in the Senior population, such terms might actually have anywhere between 5 to 15 overlapping records even though it is reported as 10, in which case, using 5 or 15 in the computations would considerably affect the odds ratios). For example, the number of significant terms (at 0.05 level after BH correction) for OR = 1/3/5/10 null levels are respectively 1858/335/105/26 if this procedure is not applied, as opposed to 1681/276/77/13 when this procedure is applied to account for the rounding (note that OR = 3/5/10 levels corresponds to the Minor/Moderate/High associated terms).
   - (2) This helps with the estimation of the effect sizes, by providing more appropriately constructed confidence intervals. As these intervals account for the additional variance due to rounding, they are necessarily wider (compared to the ones where this procedure is not applied), particularly for terms having low

number of records overlapping with IPV. For example, for “History of physical abuse”, one of the top terms in Table 1 exhibiting 10 overlapping records in SeniorIPV dataset, the 95% confidence interval for the odds ratio (i.e., raw comorbidity score) is [19.1, 218.8] if this procedure is not applied, as opposed to [10.8, 386.9] when this procedure is applied. As it can be seen, for infrequent terms such as this one, omitting this procedure results in a considerably more optimistic lower bound (19.1 as opposed 10.8) which is likely to be an overestimation. Whereas, for more frequent terms, this procedure results in a relatively minor correction: For example, “Alcohol abuse” which has 50 records in SeniorIPV dataset, has a confidence interval for the odds ratios of [10.0, 28.1] if this procedure is not applied, and [9.3, 29.9] when the procedure is applied.

Note that, to account for the effect of the rounding, we utilize the Monte-Carlo simulations based on suggestions of Reviewer 2 in the previous round, using the formulas in (https://www.sciencedirect.com/topics/mathematics/multiple-imputation) as suggested by the reviewer.

6. Is the formula in lines 162~163 the Rubin formula used to merge multiple completion data sets into a single value? As described, the formula itself does not need to be described, but a citation showing how it was conducted is required.

   Yes, this is similar to the Rubin's formula though in a different context. The use of this formula was suggested by reviewer 2 in the previous round, we added a citation for the source of this formula.

7. Line 182: The term "adjust" usually means adjusted for covariates in cohort studies. Therefore, "adjust" is confusing usage here and a more appropriate term should be used consistently. The consistent use of terminology applies to other parts of this paper as well.

   Thank you for the suggestion. In order to minimize the confusion, we revised the usage of “adjust”. We renamed the usage as “corrected” throughout the paper.

8. Line 199: Although there is a citation above, it is confusing because it suddenly appears to apply here with little explanation of the BH-procedure. What the reader needs is a general explanation of how the BH-procedure can be of benefit when applied in a case like this one. For example, what characteristics does it have compared to the Bonferroni-correction or compared to the Holms-method? (Please emphasize that the BH method allows for some familywise error rate, but suppresses beta error.) Also, Bonferroni corrects the overall type I error by using a new value of α divided by the number of repetitions of the significance-level α as a new value of α. As already mentioned, the policy of explaining in writing rathwhiter than in equations applies to the other parts of the method as well.

Thank you for giving us the opportunity to clarify this point. To avoid using an overly stringent threshold for statistical significance, we decided to use BH-procedure that bounds the false discovery rate as opposed to the family-wise error rates as in Bonferroni or Holms-procedures (i.e., we bound the average number of false discoveries, not the probability of making a false discovery). We have modified the “accounting for multiple comparisons” section to reflect this point.

9. The null hypothesis level is placed as OR=Q, but it is complicated whether it is q or Q, in addition to not defining FDR=q used in the BH-method. The authors seem to express it as Q, which later appears as μ, but the null hypothesis is only OR=1, which is less confusing. If the authors will perform this procedure, I want to see it performed with q properly defined as the null hypothesis with OR=1 after correcting for the selection bias that the authors refer to.

   Throughout the paper, we use alpha=0.05 (or as the reviewer refers to as q = 0.05) as the statistical significance level to determine the significant terms after bounding the FDR via the BH-procedure (this is fixed we always use q=0.05 level for this purpose, not to be confused with capital Q). In order to be consistent and lessen the confusion, we changed the annotation of the null hypothesis level to OR=μ* (previously it was OR = Q). Note that, we consider multiple null hypotheses. For example, we consider OR = 3 null hypothesis level to determine the Minor co-morbid terms, OR = 5 level for Moderate co-morbid terms, and OR = 10 level for High co-morbid terms. Note that, for the Minor association level, we consider OR = 3 as opposed to OR = 1 since the geometric average of the odds ratios across all terms is approximately 3 (3.16 for Senior and 3.18 for Background populations). This (choosing OR=3 opposed to OR=1 for minor association level) is done to counteract the detection bias (please see the comments #10 and #11 below for a more detailed discussion on this).

   Note that, in addition to these three null hypotheses (and three levels), during the construction of the confidence intervals that are corrected to bound the FDR of the top findings (via the procedure described in “Accounting for multiple comparisons” section), other null hypotheses OR = μ* for varying μ* (in small logarithmic intervals) are considered. **This is done so that the results are not limited to the three fixed thresholds (OR=3/5/10 for minor/moderate/high co-morbidity) and the results for other thresholds can be inferred from the confidence intervals themselves.** In these corrected confidence intervals that bound FDR, the lower bound is set to be equal to the highest μ* level a term can be deemed significant (for the FDR-adjusted significance test using BH-procedure at q=0.05 level for the corresponding OR = μ* null hypothesis). Thus, when the terms are sorted in descending order according to the lower bound of the given confidence intervals (as in the provided Tables in the manuscript), *the top terms whose lower bound is greater than μ* are necessarily the list of terms that are deemed as significant by the BH-procedure at q=0.05 level for OR = μ* null hypothesis (this interpretation is achieved by construction of the intervals).*

For example, to answer the reviewer's query, the 1681 terms (out of 2057 valid terms) that are deemed as significant by the BH-procedure at q=0.05 for null hypothesis OR = 1 in the Senior population are given by the top 1681 terms whose lower bound of the corrected confidence interval is greater than 1 in Supplementary Data 2. Similarly, the top 276 terms that are deemed as significant by the BH-procedure at q=0.05 for null hypothesis OR = 3 in the Senior population (this is the Minor co-morbidity level that accounts for the detection bias) are given in the top 276 terms whose lower bound of the corrected confidence interval for odds ratio is greater than 3 in Supplementary Data 2.

10. Line 243, I am confused because μ=3 suddenly appears. The authors should write a reason why μ=3 can be placed. If they say it is determined by geometric mean (line 287), that should be written in the method. Also, the author suddenly decides OR=3 very roughly here (line 287: approximately centered around OR=3), while the other parts are calculated using complicated methods, which is very unbalanced and confusing from the rigor of the other parts.

    To clarify this point, we added an explanation to the manuscript. This is a rounded version of the geometric mean. In the original submission, we were using the geometric mean directly and naming it as such, however upon the suggestion of the reviewers in the previous round, to avoid confusion, we decided to use a simpler threshold (a rounded down version of the geometric mean, equal to 3). This is done both because (1) we are using multiple thresholds in the revised manuscript (OR = 3/5/10 levels corresponding to Minor/Moderate/High co-morbidity), and (2) the exact geometric mean differs slightly across populations (3.16 for Senior, 3.18 for Background). Thus, to make the results easier to understand and interpret, we used the more straightforward level of 3. To reflect the reasoning behind, we added a sentence where the number 3 appears for the first time to explain our reasoning/justification for choosing 3 as the threshold.

11. The authors use the term selection bias at μ=3. Is this a selection bias in the first place? Usually in cohort studies, selection bias refers to a bias that arises at the sampling stage, such as targeting a certain biased population. in line 228: (e.g., if a severe condition is detected, more medical tests may be performed which can lead to the detection of more terms.) is not a selection bias but more an intrinsic aspect related to the use of terms. Also, if the phenomenon is caused by the mechanism as described by the authors, then the higher OR should be corrected to a greater extent and the lower OR to a lesser extent, and a method of correction such as uniformly dividing the OR would not seem to be appropriate.

    We agree with the reviewer that referring to this phenomenon as selection bias can be confusing since this is not a bias that results from a sampling stage. Thus, as suggested by the reviewer, we renamed it as detection bias.

12. Result section: Some results include matters that should be written in the Methods section, and some include subjectivity that should be discussed in the Discussion section. For example, the procedure of dividing the OR into three levels by looking at the

distribution should be indicated to the reader in advance in the method because of the procedure.

Thank you for the opportunity to work on this. We went through the manuscript again carefully, and we moved sections related to computing and analysis to methods, and we moved more subjective sections to discussion.

13. Line 295: The figure (Fig. 2) cut by OR=3/5/10 could have been drawn by simply calculating and plotting the crude OR for each word. In this case, the OR categories would be multiplied by 3 to 9/15/30....Similarly, the top 20 could have been calculated by omitting the procedure in lines 186~234. Why not just cut out the process in the middle except for the correction for multiple testing? As it stands, the correction for μ is also a parallel shift, and the effectiveness of the Monte Carlo method is still unclear. I prefer to reanalyze the data based on Occam's razor and the principle of scientific frugality. This way, the conclusion would not be so different.

    We would like to thank the reviewer for giving us a chance to clarify this point. Figure 2 is already drawn that way (they are raw/crude ORs, not comorbidities that are corrected for detection bias). Thus, the OR categories for Minor/Moderate/High co-morbidities are 3/5/10 respectively (not 9/15/30). For Minor co-morbidity, OR=3 level (as opposed OR=1) is chosen to account for the detection bias. Whereas, the procedure in lines 186-234 (in “accounting for multiple comparisons" section) is for obtaining confidence intervals (not for making statistical tests/finding significant ones for a fixed null hypothesis). As suggested by Reviewer 2 in the previous round of reviews and discussed in our response, these confidence intervals are appropriately controlled for FDR (lower bound indicating significance). We believe that this is relevant/important for ranking the terms and the framework can be applicable and helpful for the audience working with this kind of data.

14. Fig 3 & Table 1: It says "Adjusted for FDR", but isn't what is adjusted for what the authors call selection bias? Adjusting for multiple testing does not change the OR itself, only the confidence interval. Throughout, the use of the terminology should be organized.

    To avoid confusion and to make the use of terminology consistent, we now refer to these adjustments as “corrected for detection bias” and “corrected to bound the FDR” in the figure and tables.

15. Figures should exist independently as figures, and it is preferable to redefine the abbreviations used in the text with captions, etc.

    We have updated the figure captions to describe the abbreviations used.
