# Supplementary material for: Adverse health correlates of intimate partner violence against older women: Mining electronic health records
Source: PLoS One. 2023 Mar 8;18(3):e0281863. doi: 10.1371/journal.pone.0281863 (PMC9994723; doi:10.1371/journal.pone.0281863)
Supplement: Attachment — Submitted filename: Plos One referee report PONE-D-21-21436 2021 11 07.pdf [file pone.0281863.s005.pdf]

PLOS One
referee report
PONE-D-21-21436: "Identifying Adverse Health Correlates of Intimate Partner Violence against Older Women: Mining Electronic Health Records"
2021 11 07

First, note that this reviewer is a biostatistician and not a subject matter reviewer. Therefore, most of my review is concerned with the statistical methods used in the analysis.

Overview: This paper looks at the relationship between a large number of possible factors and intimate partner violence (IPV) among seniors. The data come from a large data source and the investigators identify a large number of potential factors that are looked at as possibly being related to IPV. (Initially, there are >18K factors considered.) For each examined factor, the authors basically construct a 2x2 table that is standard in epidemiological research. That is, this table looks at the cases versus the controls which are those who are and are not identified as victims of IPV. For each group (case and control), one counts the number who are exposed and not exposed to the individual factor. After creating a 2x2 table for each factor, they compute the log of the odds ratio and the associated confidence interval. If the confidence interval is greater than 0 (so the odds ratios are bigger than 1), the investigators then basically declare the factor important. Also, the reviewers compare the odds ratio for the senior population to the odds ratio of the non-senior population to see if the effect of each of the factors are discernible higher for the senior population. In the main body of the paper, the authors list some of these top factors. There is mention of a supplementary table with a larger list of the factors which are identifies as important. Note: This reviewer does note that there were some other subtle issues that are addressed in the paper. The above overview is this reviewer's understanding of the main concepts in the paper.

Overall, I like the general idea of the paper. I think that electronic health records are a largely untapped source of valuable information. However, I have important concerns that this manuscript does not do enough to account for the high number of multiple tests performed for this analysis. In the statistical literature, this is known as the multiple testing problem. Although the authors don't mention that they are doing formal hypothesis testing, the essence of this problem still applies to this study. It might seem that I am suggesting substantial changes in the analysis; however, they would probably not take long to do.

I would further note, that I did look up some papers that were produced from this general research group. Many of my below comments would have been answered in a satisfactory manner if they used the methods that they used in some other papers. I would specifically point out the paper: Karakurt, Patel, Whiting, Koyuturk, 2017, J Fam Viol 32:79-87, DOI 10.1007/s10896-016-9872-5. I will be referring to this paper as Karakurt et al in the below.

I will first list some of the statistical notes/comments/issues and then list more general matters.

1) Multiple testing. Using the criteria that a factor is acceptable in some sense (high or medium confidence) if the confidence interval is greater than 0 is basically mathematically equivalent to a null hypothesis test. (Technically, this would be a 1-way test at the .025 level.) So, the authors are doing 18k null hypothesis test (or, considering only the "valid" test, there are 2056 different test). This is the classical multiple testing problem. You are saying that something is important if the test statistic is would be rare if it was random. That is, rare means that the outcome only occurs with about a 5%

probability. Then you run this 2000 or so times and you see lots of "rare" things. This is a classic problem and there are many different ways to approach this.
a) In the other paper (Karakurt et al), a Bonferroni correction is made. So, the authors are aware of the problem. When one is doing many test, this method might be too conservative.
b) As an alternative to the Bonferroni, I prefer using the False Discovery Rate procedure (reference: Benjamini and Hochberg 1995 and Benjamini and Yekutieli 1999.) This reviewer is a fan of this method. At the end of the analysis, one gets a set of factors. In this set, it is acknowledge that some might still be by chance but overall the set contains factors which are highly likely to be important.

2) The issue that the observations are only know to the nearest "tenth". Here, the authors use a very conservative correction. When creating the log odds ratio (LOR) and the standard error estimate, they assume that the actual value is the worse possible. That is, the counts are rounded off to the nearest 10.
a) In the other paper (Karakurt et al), the authors use a Monte Carlo simulation method to sample the possible values which would be the observed value plus or minus 5. That seems very reasonable to me. Basically, this approach is a type of multiple imputation used for missing data. (Also note, the authors in that paper then compute an adjusted confidence interval. This adjusted confidence interval can be used to approximate an adjusted standard error for the LOR.)
b) In the other paper (Karakurt et al), take the sampled values are used to create an estimated confidence interval. Instead of that, one option is that one can use multiple imputation methods. That is, take the sampled values and then use multiple imputation methods to get an estimate of the standard error of the LOR which accounts for the interval censoring of the data. (see for example equations 14.7-14.10 in this article: https://www.sciencedirect.com/topics/mathematics/multiple-imputation for one reference for these formulas. These formulas are available many other places.)
c) Instead of random sampling, one can do some calculations based on the fact that one is sampling from finite discrete distribution. That is, if one is planning on sampling an integer between -5 and 5, then just calculate the LOR and standard error for each of these 11 points and then use the formulas in (b) above.

3) Prevalence: The term prevalence has a well-defined definition in the epidemiology and population health sciences. It is the number of events in a population at a designated time. There is a similar definition of the prevalence rate. The use of the term "prevalence score" to mean the log of the odds ratio is very confusing and is a misuse of well-established terms.

4) Difference between the log odds ratios between the senior population and the younger population. On page 4, the authors look at "Assessment of Differential Prevalence". They define this term in equation 5 and then give their formula for the confidence intervals in equation 6. This is a very reasonable thing to do. It is the difference between the two LOR. It is the increase in the odds between the senior population versus the non-senior population. This is a very common statistics to look at therefore the statistical properties are well known. The authors need to change the way that they compute these confidence intervals.

Note the following statistical properties: a) the LOR statistics are approximately normally distributed with the standard errors as given in the equation 2. b) Since the senior population and the younger population are completely different populations, then these samples are statistically independence. c) Therefore, the difference of the two statistic defined in equation 5 has an approximate a normal distribution. d) standard error of difference of between the sampled log odd ratio's between these two populations is just "square root of the sum of the squared standard errors". That is, if the standard

errors are S1 and S2, then the standard error of the differences is sqrt(S1^2 + S2^2). One can then get the confidence interval of the difference in the log odds ratio by the methods already discussed in the paper. (That is, it is the difference +/- 1.96* standard error of the difference as described in equation 3).

5) I don't see what the value of equation 4. That is, the value of using a mean adjusted prevalence score which is obtained by subtracting the LOR by the average of the LOR and then using that to declare "high" effect. That seems like an attempt to control for the multiple testing, but does not appear to have any validity in controlling for the multiple testing problem. If one took completely random data and then applied this procedure, it would falsely show that some of these random statistics showed a high effect. In fact, since among a random sample of values, by construction, there will always be some that would be "above the average". (Aside: you did not describe how to construct the confidence interval associated to this mean adjusted prevalence score.) Maybe I'm missing something here and there is validity to compute the mean adjusted prevalence score. If so, then please provide some literature to support this method.

Some more general comments about the paper:

6) I don't know what the source of the data is. I am not researcher working in USA, so I am not familiar with this data source.
a) this data source should be reference and either described or a paper which adequately describes this data source should be reference.
b) I don't know where these terms/factors come from. It looks there is a query to the data set program, but I don't know what kinds of queries are being made. I am familiar with ICD codes and know where they come from, but I don't know why or how the authors came up with 18K different factors that were then tested.

7) The type of results reported. I think that if the purpose of this paper is to give information on the factors which contribute to IPV in seniors, then I think more analysis should be done. In the results section of the paper, the authors report that they identified 250 and 1240 terms with high or medium confidence. Then, in tables 1 and 2, they list the top 20 terms. (I did not receive the supplemental file, but I presume the other 1000+ terms are listed there.) My main concern is, "how is a scientist suppose to synthesis this information?" These are a lot of terms here. I would suggest that this information would be more useful if this paper was able to organize or structure this information better. For example, they could cluster the subjects or group together the main terms in some way. (A first pass cluster analysis or factor analysis for example.) I would assume that there is high correlation between these terms. Note that there is a total of only 420 senior IPV subjects. (See Figure 1b) Also, I see that members of this group have done similar work before. (See for example, Hacialiefeniouglu et al, 2021, Scientific Report.)

8) The statistics that are reported in tables 1 and 2. In these tables, the authors report log (base 2) odds ratios and counts. These are not the easiest values to interpret. Usually, one would transform the log odds ratio and confidence interval to the odd ratio scale. The usual population scientist thinks in terms of odds ratios. Most cannot convert from log odds ratios in their heads. (And even the ones that do have a feel for log odds ratios usually works in the natural log scale not in base 2. So, they would have to multiple their usual calibrated LOR by 0.7 to make the conversion.) The raw counts are okay, but the proportions are more convenient for those who want to interpret the results.
